# Recurrent Dislocation of the Patella in Kabuki Make-Up Syndrome

**DOI:** 10.1155/2012/501453

**Published:** 2012-12-18

**Authors:** Lucie Rouffiange, Jean-Paul Dusabe, Pierre-Louis Docquier

**Affiliations:** Department of Orthopaedic Surgery, Cliniques Universitaires Saint-Luc, 10 Avenue Hippocrate, 1200 Brussels, Belgium

## Abstract

Two patients with Kabuki make-up syndrome with bilateral recurrent dislocation of the patella are presented. They had generalized ligamentous laxity and patellofemoral dysplasia. Both developed patellar dislocation in adolescence and required surgery, with medial transfer of the tibial tuberosity associated with vastus medialis plasty (Insall technique). One postoperative complication occurred in one case: a nondisplaced tibia fracture at the sixth postoperative week that healed with conservative means. Final results were good in both cases. Good surgical results can be achieved in patellar dislocation in patients with Kabuki syndrome.

## 1. Introduction

The Kabuki make-up syndrome (KMS) is a rare congenital syndrome that was first reported in Japan, and later in other ethnic groups and in Europe. It is a syndrome with distinctive facial features, skeletal anomalies, dermatoglyphic abnormalities, mild or moderate mental retardation, and postnatal deficiency. The main skeletal anomalies first reported were vertebral anomalies [1–7], hand abnormalities [1–7], and hip dislocation [8]. More recently, clavicle pseudarthrosis [9], clubfeet [10], and patellar dislocation [11–13] were also described. Only few cases of surgery for patellar dislocation have been reported. We describe two cases with recurrent dislocation of the patella in which surgery permitted to obtain stable patella. 

The patient and her family were informed that data concerning the case would be submitted for publication.

## 2. Case Presentation

**Case 1**

This young patient was a second born to nonconsanguineous healthy parents. He had global developmental delay with sitting position acquisition at 18 months of age, walking at 3 years, and first language acquisition at 4 years. He had mental retardation (total QI 64). He was diagnosed as KMS at the age of 6 years. He had facial dysmorphism with wide forehead, hypertelorism, almond eyes, large and poorly hemmed ears, and thin upper lips with drooping commissures. He had several other malformations as cryptorchidism, short penis, joint hyperlaxity, fifth finger clinodactyly, and widening of the second phalanx of the thumb. He was also obese and had skin areas of hypo- and hyperpigmentation. He had chronic otitis media complicated with tympanosclerosis.

He had long surgical history. He was operated for a chalazion, and for adenotonsillectomy at the age of 3 and 4 years, respectively. At the age of 6 years, he was operated on for bilateral cryptorchidism and for tympanoplasty. Multiple other surgeries were performed for tympanosclerosis and cholesteatoma and hearing loss. 

**Figure 1:**
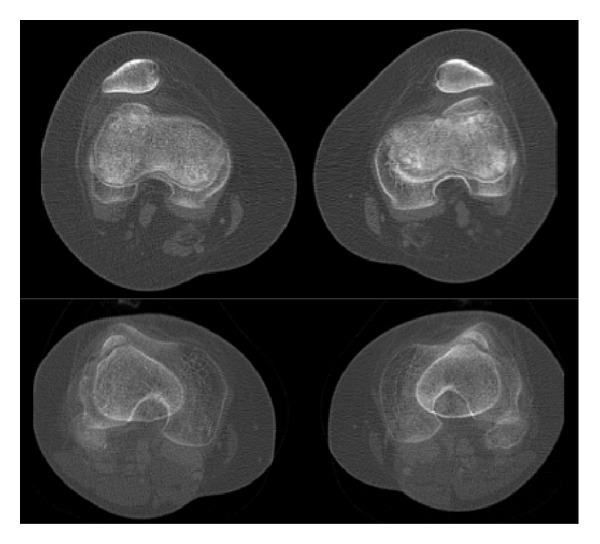
Top: CT-scan of both knees performed at the age of 15 years, showing bilateral patellofemoral dysplasia and permanent subluxation of patella. Bottom: distance TT-TG (tibial tubercle-trochlear groove) of 19 and 27 mm at the left and right side, respectively.

**Figure 2:**
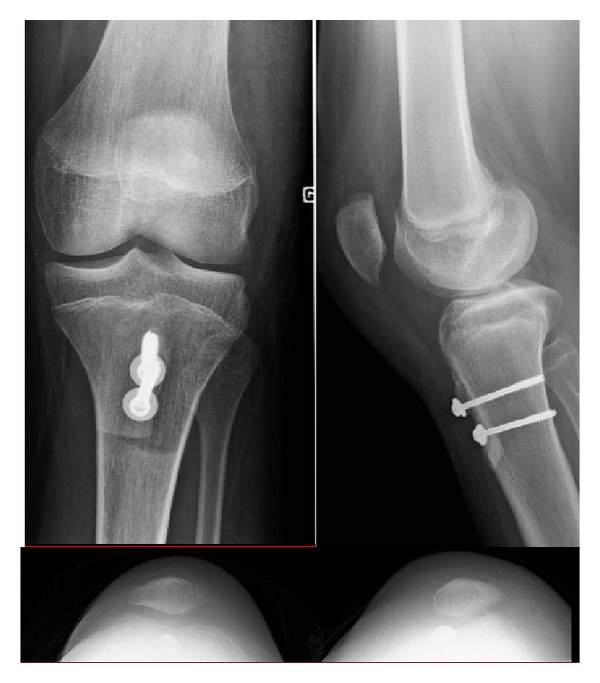
Postoperative radiograph of the knee after medial tibial tubercle transfer and vastus medialis plasty. The patella is better centred.

At the orthopaedic point of view, he had asymptomatic flatfeet and patellar instability with frequent subluxations and dislocations. CT-scan evaluation showed femoropatellar dysplasia with a distance TT-TG (tibial tubercle-trochlear groove) of 19 and 27 mm at the left and right side, respectively (Figure 1). Conservative treatment was first attempted with physiotherapy, stabilizing brace, and sport abstention. Despite 2 years of conservative treatment, femoropatellar subluxation episodes occurred daily and true dislocations every 3 months. The knees were painful, and it was decided to perform a stabilizing surgery. The right knee was operated on at the age of 17 years and 11 months with medial transfer of tibial tubercle, lateral release, and plasty of the vastus medialis (Insall technique) (Figure 2). The knee was immobilized with a brace in extension for 6 weeks with weight bearing allowed. Physiotherapy was allowed after 6 weeks. The left knee was operated with the same procedure at the age of 18 years and 3 months. A nondisplaced fracture of the tibia diaphysis occurred at the sixth postoperative week due to a drop during reeducation. The fracture healed with 3 weeks of casting and 3 weeks of bracing. Partial weight bearing was allowed 3 weeks after the fracture. At the first postoperative year he had regained full mobility of the knees with disappearance of the pain.

**Case 2**

This young girl was born to nonconsanguineous healthy parents. She had global developmental delay. She had mental retardation. She had facial dysmorphism and cleft palate that was operated on. She was also operated on for dentomaxillar dysharmonia with maxillary osteotomy and for tympanoplasty.

**Figure 3:**
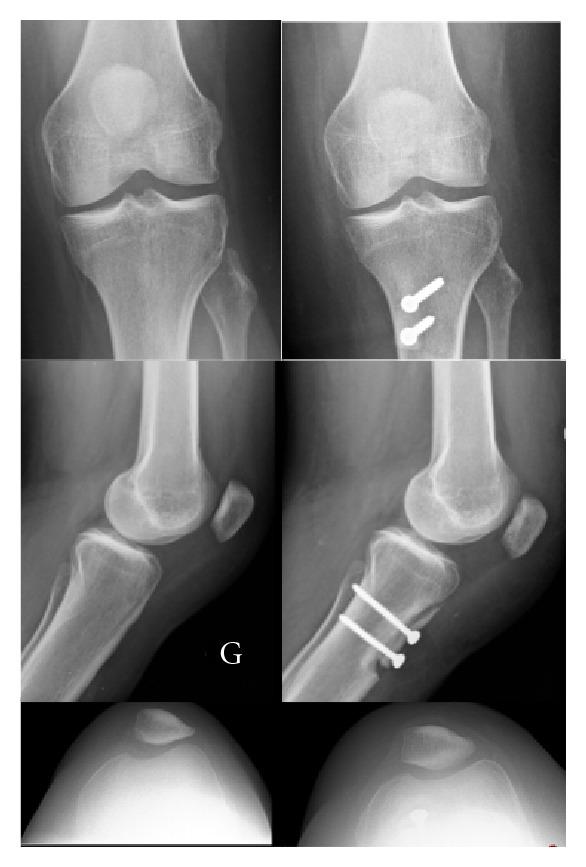
Pre- (left) and postoperative (right) radiograph of the left knee after medial and distal tibial tubercle transfer and vastus medialis plasty. The patella is better centred.

At the orthopaedic point of view, she had symptomatic bilateral hallux valgus that was operated on at the age of 16 years. She had also patellar instability with frequent subluxations and dislocations. CT-scan evaluation showed femoropatellar dysplasia with a distance TT-TG of 23 and 17 mm at the left and right side, respectively. Conservative treatment was first attempted with physiotherapy, stabilizing brace, and sport abstention, but instability was still present and the knees were painful, and it was decided to perform a stabilizing surgery. The right knee was operated on at the age of 16 years and the left one at the age of 22 years. Surgical procedure consisted of medial transfer of tibial tubercle associated with lateral release and vastus medialis plasty (Insall technique) (Figure 3). The knee was immobilized with a brace in extension for 6 weeks with weight bearing allowed. Physiotherapy was allowed after 6 weeks. No complications were encountered, and the patient regained full mobility of the knees at the first postoperative year.

## 3. Discussion

Kabuki syndrome, Kabuki make-up syndrome, or Niikawa-Kuroki syndrome was first described simultaneously and separately by two Japanese authors: Niikawa et al. [5] and Kuroki et al. [3]. The syndrome is so named because affected children have facial resemblance to the actors in the traditional Japanese theatre (Kabuki). There is no firm evidence for any specific chromosomal abnormality [10]. More recently, mutations in MLL2 were demonstrated as a major cause of Kabuki syndrome [14].

 There are 5 cardinal features [1, 2, 4–7]. The main feature is the facial dysmorphism (long palpebral fissures with eversion of the lateral one-third of the lower eyelid, arched eyebrows with sparseness of the lateral one-third, short nasal columella with depressed nasal tip, and prominent or cupped ears) [5]. Other features are developmental delay or mental retardation, postnatal short stature, skeletal anomalies, and dermatoglyphic anomalies [3, 9]. Other possible clinical manifestations of KMS are cardiac anomalies, deafness, ophthalmologic anomalies, missing teeth, frequent infection, cleft palate, intestinal malrotation, anorectal anomalies, seizures, and endocrine anomalies [1, 3, 5, 15, 16]. 

Skeletal anomalies can consist of brachydactyly and clinodactyly of the fifth finger [1–7], scoliosis and vertebral malformations [1–7], clavicle pseudarthrosis [9], clubfeet [10], ligamentous hyperlaxity, hip dislocation [8], and patellar dislocation [11–13].

Only five cases of surgery for patellar dislocation in KMS have been reported in the literature. The first report is the one of Niikawa et al. [4]. Ikegawa et al. [12] described 3 cases with recurrent dislocation of the patella developed in adolescence and with generalized ligamentous laxity. Surgery was performed in two: Elmslie-Trillat operation in the first and Campbell operation in the second. There was good result in the first case and recurrence in the other. The third patient had cardiac anomalies precluding operation. Another operated case is reported by Burke and James [11]. Kurosawa et al. [13] reported four cases of patellar dislocation with only one operated with good results. Surgery consisted of resection of free patellar fragment and tibial tubercle transfer. In our 2 patients, the treatment was first conservative but without good results. The surgical treatment improved the 2 patients. A summary of all the reported cases in the literature is given in Table 1. Six cases were surgically treated with success in five. The cases with conservative treatment were also improved. 

In conclusion, in case of patellar dislocation in KMS, surgical procedure can be attempted after failure of conservative method. Good results can be achieved with tibial tubercle transfer associated with vastus medialis plasty.

## Figures and Tables

**Table 1:** Summary of the reported KMS cases with surgically treated patellar dislocation.

Author	Sex	Age at the time of the first patellar dislocation (years)	Side	Surgery type	Evolution	Hyperlaxity	Other skeletal anomalies	Obesity
Niikawa et al. (1988) [4]								
	F	17	Left	Not precise	?	?	Short metacarpals	?

Ikegawa et al. (1993) [12]								
Case 1	F	12	Right	Elmslie-Trillat operation	Improvement	+	Acetabular dysplasia	−
Case 2	F	8	Right	Campbell operation	Recurrence	+	Genu valgum	?

Burke and Jones (1995) [12]								
	F	2	Bilateral	Not precise	Knee contracture persistence	?	None	−

Kurosawa et al. (2002) [13]								
	M	16	Right	Resection of patellar fragment and tibial tubercle transfer	Improvement	+	None	−

Our study								
Case 1	M	13	Bilateral	Tibial tubercle transfer and vastus medialis plasty	Improvement	+	5th finger clinodactyly, widening of the 2nd thumb phalanx	+
Case 2	F	10	Bilateral	Tibial tubercle transfer and vastus medialis plasty	Improvement	+	None	−
